# Object recognition via echoes: quantifying the crossmodal transfer of three-dimensional shape information between echolocation, vision, and haptics

**DOI:** 10.3389/fnins.2024.1288635

**Published:** 2024-02-19

**Authors:** Santani Teng, Caroline Danforth, Nickolas Paternoster, Michael Ezeana, Amrita Puri

**Affiliations:** ^1^Smith-Kettlewell Eye Research Institute, San Francisco, CA, United States; ^2^Department of Biology, University of Central Arkansas, Conway, AR, United States; ^3^Department of Psychology, Vanderbilt University, Nashville, TN, United States; ^4^Department of Psychology, Cornell University, Ithaca, NY, United States; ^5^Georgetown University School of Medicine, Washington, DC, United States

**Keywords:** echolocation, blindness, object perception, crossmodal perception, spatial resolution

## Abstract

Active echolocation allows blind individuals to explore their surroundings via self-generated sounds, similarly to dolphins and other echolocating animals. Echolocators emit sounds, such as finger snaps or mouth clicks, and parse the returning echoes for information about their surroundings, including the location, size, and material composition of objects. Because a crucial function of perceiving objects is to enable effective interaction with them, it is important to understand the degree to which three-dimensional shape information extracted from object echoes is useful in the context of other modalities such as haptics or vision. Here, we investigated the resolution of crossmodal transfer of object-level information between acoustic echoes and other senses. First, in a delayed match-to-sample task, blind expert echolocators and sighted control participants inspected common (everyday) and novel target objects using echolocation, then distinguished the target object from a distractor using only haptic information. For blind participants, discrimination accuracy was overall above chance and similar for both common and novel objects, whereas as a group, sighted participants performed above chance for the common, but not novel objects, suggesting that some coarse object information (a) is available to both expert blind and novice sighted echolocators, (b) transfers from auditory to haptic modalities, and (c) may be facilitated by prior object familiarity and/or material differences, particularly for novice echolocators. Next, to estimate an equivalent resolution in visual terms, we briefly presented blurred images of the novel stimuli to sighted participants (*N* = 22), who then performed the same haptic discrimination task. We found that visuo-haptic discrimination performance approximately matched echo-haptic discrimination for a Gaussian blur kernel σ of ~2.5°. In this way, by matching visual and echo-based contributions to object discrimination, we can estimate the quality of echoacoustic information that transfers to other sensory modalities, predict theoretical bounds on perception, and inform the design of assistive techniques and technology available for blind individuals.

## Introduction

The perception of objects, among the most crucial functions of sensory processing, is a multi-modal and crossmodal phenomenon. In the absence or insufficiency of vision, many organisms employ *active echolocation* by perceiving cues embedded in self-generated acoustic reflections in order to obtain information about objects in their environment. Best known in bats and dolphins, echolocation is also practiced as a perceptual aid by some blind humans (Kolarik et al., 2014; Thaler and Goodale, 2016), who have refined a method of producing tongue “clicks” to produce consistent, well characterized echoes (Rojas et al., 2009; Thaler et al., 2017). This allows proficient echolocators, and in some cases even novices, to perceive the presence, positions, and sizes of reflecting objects (Rice, 1967; Teng and Whitney, 2011; Schörnich et al., 2012; Teng et al., 2012), their orientation relative to nearby features (Rosenblum et al., 2000; Dodsworth et al., 2020), and the general scale of their surroundings (Flanagin et al., 2017). In contrast to non-echolocating blind controls, blind echolocators navigate obstacles more rapidly (Thaler et al., 2020), spatially bisect auditory space more effectively (Gori et al., 2014; Vercillo et al., 2015), and experience “visual” perceptual phenomena such as size constancy (Milne et al., 2015a) and the size-weight illusion (Buckingham et al., 2015).

In contrast to stimulus localization, it is less straightforward how object perception arises from the complex pattern of echoes returning from surfaces with widely varying sizes, shapes, poses, and material properties. Yet many biological echolocators routinely acquire detailed object information from echoes. Bats distinguish small spheres from mealworms (Simmons and Chen, 1989), pollinated from virgin flowers (Von Helversen and von Helversen, 1999; Simon et al., 2011), and size-invariant object categories (Genzel and Wiegrebe, 2013), relying on the fine spectrotemporal structure of the echo returns to compute “acoustic images” (Simmons, 1989), match acoustic templates (Vanderelst et al., 2016), and integrate echoic and visual representations of learned objects (Danilovich and Yovel, 2019). Dolphins similarly discriminate object echoes, integrating across senses—e.g., in visual-echoic match-to-sample tasks—without first learning an associative pairing (Pack and Herman, 1995), independently of reward structure (Harley et al., 2003). This suggests that the encoded object representations in these animals are crossmodally transferable, rather than standalone arbitrary acoustic templates, and fine-grained enough to support visual or echoacoustic comparison.

In humans, echolocation-based object recognition is far less well understood, although both blind and sighted individuals have demonstrated some ability to use echoes to discriminate material textures (Hausfeld et al., 1982; Milne et al., 2015b; Sumiya et al., 2019) and gross object or surface shape (Thaler et al., 2011; Arnott et al., 2013; Milne et al., 2014; Sumiya et al., 2019), with down-pitched artificial ultrasonic echoes potentially facilitating performance (Sumiya et al., 2019; Fujitsuka et al., 2021). Because these tasks probed unisensory echoacoustic discrimination, it remains largely unknown how this echoic object information is encoded, e.g., as a strictly echoacoustic representation or a more abstracted representation useful in real-world multisensory scenarios (e.g., echolocating an object to grasp or move it).

To address this question, here we used a crossmodal match-to-sample paradigm to investigate the capacity of human observers to extract and transfer object shape information across echolocation and haptics (Experiment 1) and vision and haptics (Experiment 2). We hypothesized that a trained human echolocator could, in principle, use echolocation to recognize the structure of three-dimensional objects well enough to then discriminate them haptically, implying a crossmodally accessible representation. Among the subpopulation of blind persons, very few are known expert echolocators, and it is unclear how or even whether echoes give rise to crossmodally transferable object-level percepts in humans as has been shown in dolphins (Pack and Herman, 1995; Harley et al., 2003). Thus, we adopted a single-observer approach in Experiment 1, treating participants as individual case studies to probe for crossmodal echo-haptic transfer. Further, in Experiment 2, we sought to quantify the resolution of echo-haptic object information transfer in terms of *crossmodal equivalent blur*, using an approach based on the principle that the more sharply an object is represented in one modality, the more discriminable the object should be in a different modality. By briefly presenting images of the objects that had been blurred to varying degrees, and measuring performance on a visual-haptic match-to-sample task, we aimed to determine the level of blur producing equal performance to the echo-haptic task.

## General method

Our first aim was to assess whether three-dimensional object shape information acquired via echolocation could be transferred between sensory modalities to aid in haptic object perception. Next, we determined the degree of object image blur that would lead to equivalent performance on an analogous visuo-haptic discrimination task. In both experiments reported here, we applied an ABX match-to-sample paradigm in which on each trial, participants first inspected a target object using either echolocation (Experiment 1) or vision (Experiment 2). The target object was then removed, and replaced by two physical objects, the target and a distractor object, placed side-by-side. The target location (to the participant’s left or right side) was randomized. The matching phase of each trial involved inspecting the target and distractor object pairs using only touch, and reporting which of the two objects was the target that had just been presented for echoic or visual sampling.

## Experiment 1: echo-haptic matching

### Experiment 1 methods

#### Participants

Five blind expert echolocators and 13 sighted controls, all of whom were naive to the task, participated in this experiment. Of these, all five blind practitioners and eight sighted controls performed in both experiments. Two participants only echolocated Common objects for a total of 10 in Experiment 1A; three only echolocated Novel objects for a total of 11 in Experiment 1B. The sighted controls (5 male, mean age 22.6 y) reported normal or corrected-to-normal vision and hearing. Blind participants (5 male, mean age 27.3 y; see Table 1) were selected for total or near-total blindness and self-reported long-term, frequent use of active tongue-click echolocation totaling ≥10,000 h estimated experience (see also Teng et al., 2012). Participants provided informed consent in accordance with guidelines set forth by the UC Berkeley and Smith-Kettlewell Eye Research Institute Institutional Review Boards, and were compensated for their time. To mitigate barriers to participation involving long-distance travel to our lab, we tested blind echolocators at a remote testing site that was readily accessible to them, with similar acoustic conditions (quiet office/living room environment) to our lab in which sighted participants were tested.

**Table 1:** Blind echolocating participants.

ID	Symbol	Blindness onset age (y)	Age at test (y)	Blindness severity	Blindness etiology
B1	⬤	0	28	Total	Glaucoma
B2	◼	5	25	LP	Retinitis Pigmentosa, Juvenile Macular Degeneration
B3	▲	14	27	Total	Optic Nerve Atrophy
B4	▼	1.8	33	Total	Retinoblastoma, Enucleation
B5	⬥	17	22	Total	Familial exudative vitreoretinopathy

Symbols correspond to graphic labels in Figures 2, 3. LP, light perception only; no spatial vision.

#### Stimuli

**Figure 1:**
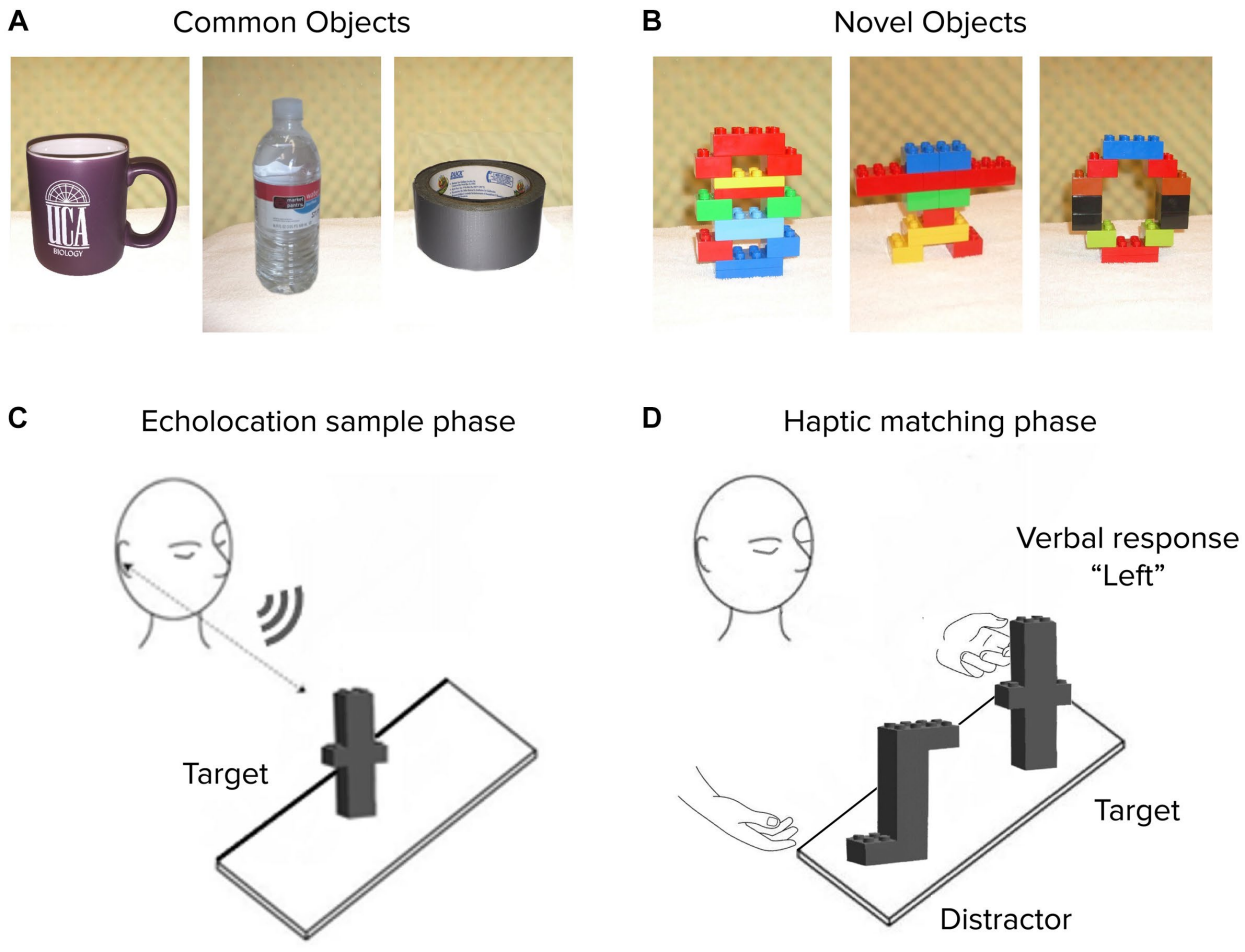
Experiment 1 stimuli and procedure. Representative objects are shown from Common **(A)** and Novel **(B)** sets. On each trial, an untimed echolocation sampling phase **(C)** was followed by a haptic matching phase **(D)** for both blind and sighted subjects (all of whom were blindfolded at all times). Verbal response identified the target on the “*Left*” or “*Right*”.

Stimuli comprised two sets of 16 distinct objects each, tested separately. Experiment 1A involved *Common* objects: everyday items varying in size, shape, weight, surface material, and composition. The objects were roughly handheld in size, such as a water bottle, a coffee mug, and a roll of packing tape, similar to stimuli distinguished crossmodally by dolphins (Harley et al., 2003). In Experiment 1B, stimuli comprised 16 *Novel* objects, constructed using LEGO^®^ DUPLO^®^ blocks in varying configurations but identical weight, material, and total solid volume across objects (Figure 1). Thus, object differences were restricted to the configural (i.e., shape) dimension and everyday object familiarity was eliminated as a potential cue. Images depicting the full common and novel stimulus sets are included in an appendix.

#### Procedure

We used a crossmodal echo-haptic match-to-sample paradigm, well suited for stimuli differing along multiple complex dimensions (Hautus and Meng, 2002; Macmillan and Douglas Creelman, 2004), and adapted from previous echolocation studies with dolphins (Pack and Herman, 1995; Harley et al., 2003). Each trial in this task had two phases: an echolocation sample phase and a haptic match phase (Figures 1C,D).

In the sample phase, untimed but typically lasting ~30–90 s, participants examined a target object on a padded tabletop surface using only tongue-click echolocation. They were not allowed to touch the object, or to cross the vertical plane of the nearest tabletop edge, but were otherwise free to vary the angle at which they ensonified the target with tongue clicks. While we did not systematically track this behavior, movements typically comprised translations up to about one-half body width in either azimuthal direction and half a head height vertically—i.e., constraints of comfortable motion while seated in a chair. Based on a subset of monitored trials, expert echolocators typically generated around 15–20 clicks per trial, compared to about 60 for sighted controls. To minimize incidental cueing from experimenter movement, object placement, or ambient sound, the tabletop and nearby working surfaces were padded with towels, and the experimental setup was backed by a semicircular sound-dampening foam surface extending 1–2 m above the table surface. All participants, blind and sighted, wore blindfolds at all times to account for residual vision as well as equate any tactile or auditory effects of wearing a wraparound eye cover.

The target object was then briefly removed and then reintroduced on the table along with a distractor object, an action that consistently lasted ~5 s. In this haptic match phase, the target was placed with the same face toward the participant as in the sample phase. Participants used their hands to freely inspect the target and distractor with either or both hands from any angle, but not to pick them up, nor to re-inspect them echoacoustically. The target object was identified via verbal report (Figure 1D). No feedback was provided.

Target and distractor locations for each trial were pre-randomized and counterbalanced, while the sample-to-match placement order was consistent, ensuring that experimenter actions did not serve as informative cues to the target. To minimize the likelihood of learning arbitrary pairwise associations within the practical constraints of our object set (and limitations on participant access and available experiment time), we presented each of the 16 objects per set as a target three times, resulting in 48 trials for each of the two sets. On each trial, the target was paired with a random distractor, with each object appearing as a distractor three times within a session. The order of Experiments 1A and 1B was counterbalanced across participants.

#### Analysis

To estimate group-level ability to transfer echo object information across modalities, we compared each of the four subgroups (Blind/Sighted, Common/Novel) independently against a chance performance level of 50% correct using a 1-sample t-test, alpha = 0.05. These and other statistical results are presented below both uncorrected, and corrected for multiple comparisons using the Benjamini-Hochberg procedure at a false discovery rate *q* of 0.05 (Benjamini and Hochberg, 1995). Additionally, for the subset of participants who completed both object conditions, we conducted a 2 × 2 repeated-measures ANOVA with group (Blind/Sighted) as between-subjects factor and object category (Common/Novel) as within-subjects factor, seeking to determine whether performance was significantly dependent upon those participant or stimulus attributes.

Further, echolocation ability exhibits strong individual differences (Teng and Whitney, 2011; Milne et al., 2014; Norman et al., 2021), and echo-haptic crossmodal transfer of echoic object information has not previously been systematically explored in human observers. Thus, in addition to group analyses, we treated participants as individual independent cases by assessing performance relative to chance (50% correct, or 24 out of 48 trials per session) with an exact binomial test. For each participant, significance *p_i_* was operationalized as the conditional probability of achieving at least the given success rate under the null hypothesis of no echo-haptic crossmodal transfer. Scores were also Z-transformed relative to chance. Next, using a combined probabilities approach adapted from clinical meta-analyses, we pooled the individual *p*-values for blind and sighted participants under each condition according to Fisher’s method (Elston, 1991; Fisher, 1992) to evaluate the significance of a single chi-squared statistic for *k* combined values:


$$X_{2k}^{2}=-2\overset{k}{\underset{i=1}{\sum }}\ln\;p_{i}$$

Additionally, we combined Z-scores using Stouffer’s method, which accounts for the directionality of the alternative hypothesis and is more robust to outlying small p-values (Stouffer et al., 1949; Mosteller and Bush, 1954; Whitlock, 2005):


$$Z_{\mathrm{Stouffer}}=\frac{\sum_{i=1}^{k}Z_{i}}{\sqrt{k}}$$

The resulting test statistics, p_Fisher_ and Z_Stouffer_ (p_F_, Z_S_), represent estimated probabilities of observed performance under the null hypothesis for every individual, or inversely, a measure of support for the alternative hypothesis in at least one individual.

Finally, in a post-hoc exploratory analysis, we sought to determine whether certain objects disproportionately drove performance. Our experimental design did not exhaustively pair every object-as-target repeatedly with every object-as-distractor, both due to practical constraints and because we wished to avoid facilitating gradually learned arbitrary echo-haptic associations. Still, to examine whether stimulus-level patterns arise in the aggregate, we computed confusion matrices and summed them across participants for each of the four subgroups. Correct responses per object are represented on the diagonals, and false alarms (incorrectly selecting a given object as matching the sample) are represented by the summed off-diagonal column frequencies. Both hits and false alarm frequencies across the 16 objects were compared via chi-square tests against a null hypothesis distribution, i.e., that objects would be selected, correctly or incorrectly, with equal frequency.

### Experiment 1 results and discussion

**Figure 2:**
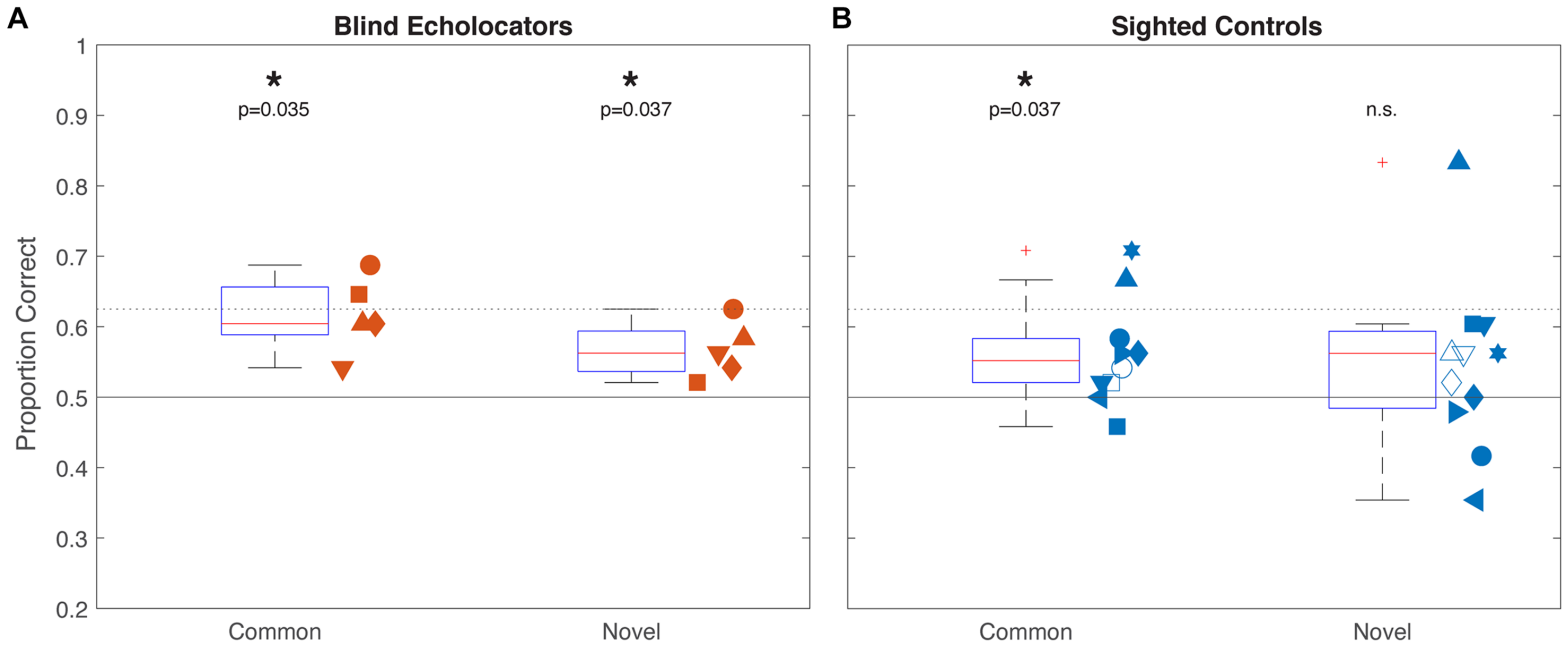
Experiment 1 group results for Blind (**A**; orange) and Sighted (**B**; blue) participants. Boxplots indicate median, 25th and 75th percentiles, and non-outlier range. Pluses outside whiskers indicate outliers. Horizontal rules indicate chance (50%) and individual-level above-chance performance threshold (62.5%) by binomial test. Significance indicators at top reflect 1-sample t-tests against chance, corrected for multiple comparisons. Adjacent scatterplots indicate individual participants’ performance with unique shape within each group, with x-position jittered for visibility. Open shapes indicate participants who only completed a session in one condition; all others performed both Common and Novel conditions.

At the group level, blind echolocators (Figure 2A) correctly matched echolocated samples to haptic targets with above-chance accuracy for Common (61.7% mean; 95% confidence interval 54.9–68.4%; 1-sample t-test vs. 50%, *p* = 0.009; *p* = 0.035 corrected) as well as Novel [56.7% (51.7–61.6%); *p* = 0.0205; *p* = 0.037 corrected] object types. Sighted controls (Figure 2B) performed more variably, with above-chance accuracy for Common: 57.0% (50.8–61.6%; *p* = 0.028; *p* = 0.037 corrected) but not for Novel: 54.4% (46.3–62.8%; *p* = 0.248) objects. We next analyzed the data from the five Blind and eight Sighted participants who completed sessions for both object types using a 2 × 2 ANOVA with object type (Common/Novel) as a within-groups factor and vision status (Blind/Sighted) as a between groups factor. There were no significant main effects of object type [*F*(1,11) = 1.36, *p* = 0.687] or group [*F*(1,11) = 0.55, *p* = 0.473], or any interaction between factors [*F*(1,11) = 0.135, *p* = 0.720].

**Figure 3:**
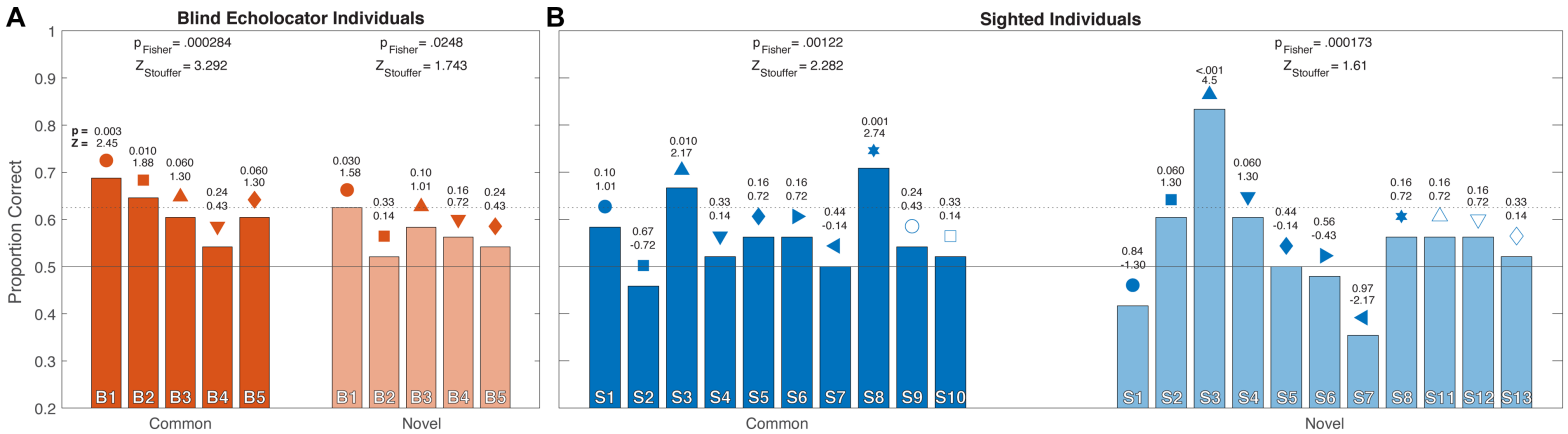
Experiment 1 individual results for Blind (**A**; orange) and Sighted (**B**; blue) participants. As in Figure 2, horizontal rules indicate chance (50%) and individual above-chance performance threshold (62.5%) by binomial test, and individual participants are identified with unique symbols above each column. For clarity, columns depicting Novel data are desaturated compared to Common data. Individual p and Z score estimates are indicated immediately above each column (Z_critical_ = 1.645). Combined test statistics p_Fisher_ and Z_Stouffer_ are shown above grouped columns for each condition, corrected for multiple comparisons (see Methods).

In the individual analysis, every blind echolocator (orange; Figure 3A) haptically discriminated Common and Novel objects sampled echoically with greater than 50% accuracy. One individual performed significantly above chance for both Common and Novel objects, while another performed above chance for Common objects only. Combined probabilities for the blind participants were significant for both Common (p_F,Common_ = 0.00014; 0.00028 corrected; Z_S,Common_ = 3.29) and Novel (p_F,Novel_ = 0.025 corrected; Z_S,Novel_ = 1.74) objects. Among the sighted participants (blue; Figure 3B), one of 10 participants scored slightly below 50% for Common objects; the rest performed at or above 50%, two significantly so. One of these participants (upright filled triangle in Figures 2B, 3B) was also the only one to score significantly above 50% for Novel objects. Combined probabilities for sighted observers were significant for Common (p_F,Common_ = 0.00092; 0.0012 corrected; Z_S,Common_ = 2.28) objects; for Novel objects, the combined probability approaches yielded diverging results (p_F,Novel_ < 0.000043; 0.00017 corrected; Z_S,Novel_ = 1.61). The lower Z-scores relative to value of ps reflect the greater variability across participants, including scores below 50%. Overall, the combined probability tests indicated that for both object conditions, the observed performance across blind individuals was unlikely to be due to chance, consistent with the group analyses. Combined probabilities for sighted individuals similarly indicated significantly above-chance performance for Common objects, but diverged for the Novel condition, with a significant p_Fisher_ but Z_Stouffer_ below the critical value of 1.645.

**Figure 4:**
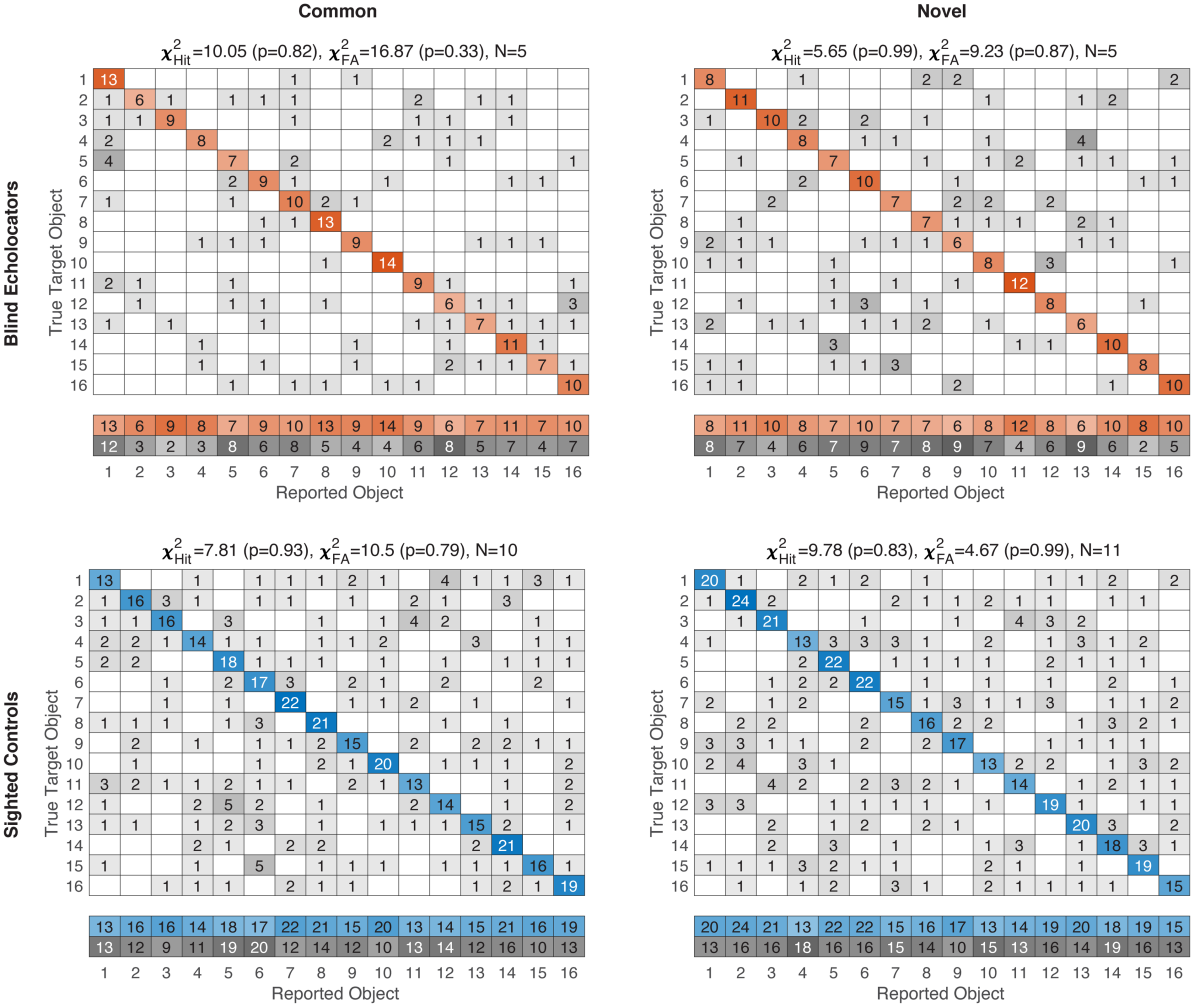
Item-level confusion matrices for Experiment 1. Blind/Sighted groups color-coded as in previous figures. Saturation level of colored (correct) or grayscale (incorrect) cells indexes pooled frequencies. For clarity, Hit and False Alarm (FA) response distributions are in the summary rows below each matrix. Corresponding chi-square test results against null hypotheses of uniformity are displayed above each subplot, along with pooled sample sizes. No group-condition combination rejected the null hypothesis, suggesting responses were not strongly driven by individually confusable items. Object IDs are detailed in supplemental data.

For both Common and Novel object classes, the response distributions pooled across participants did not differ significantly from uniformity for either group or object category (Figure 4; all chi-square results *p* ≥ 0.33, all *df* = 15). Thus, we did not find evidence to suggest that particular objects were selected disproportionately compared to the others, either as a correct match or as an incorrect false alarm.

Taken together, these results suggest that 3D objects sampled by echolocation can be discriminated to some extent, and that this shape information is available across sensory modalities. Blind echolocators performed more consistently as a group, with all five participants performing echo-haptic discrimination above 50% in both categories. By contrast, at least two sighted participants scored at or below 50% in each object category. Sighted group and individual analyses suggested significantly above-chance discrimination performance for Common objects; however, for Novel objects, despite some individual high performances and a significant Fisher’s value of p, neither t-test or Stouffer’s Z results rejected the null hypothesis of chance-level performance.

Overall, the task was quite difficult, judging by participants’ (informal) reactions and relatively low overall performance. We also did not find significant item-level patterns arising from the pooled confusion matrices. This may stem from the relatively small object size, from the larger object set, and from a task design that intentionally minimized pre-testing practice and stimulus repetition, all in contrast to previous echoacoustic object discrimination tasks (Arnott et al., 2013; Milne et al., 2014). In addition, the crossmodal (echo-haptic vs. strictly echoacoustic) nature of the experiment likely increased difficulty relative to a comparable unimodal task (Newell et al., 2005; Ernst et al., 2007).

Thus, it is clear that the echo-haptic task in Experiment 1 was challenging for both sighted individuals without previous experience using EL and blind expert echolocators, though the expert participants performed above chance more consistently than sighted controls. In light of previous work relating the spatial resolutions of echoacoustic and visual object localization (Teng et al., 2012), we considered that spatial resolution may similarly constrain the crossmodal transfer of active echolocation-based object information. In other words, representations of our stimulus objects may effectively be “blurred” by factors such as human-audible echoacoustics (frequencies typically spanning wavelengths around 2–20 cm), the limits of echoacoustic perceptual processing, and the crossmodal transfer required by our task.

Accordingly, we designed a second experiment in which sighted participants completed an analogous crossmodal task, but rather than having observers use echoes to examine target objects, we presented the samples in visual form, while systematically controlling the spatial resolution of the images using a blurring kernel.

## Experiment 2: visuo-haptic matching

In Experiment 2, we aimed to quantify the echoic representation underlying haptic discrimination performance in Experiment 1 by assessing the equivalent visual resolution of the transferred information. As object discrimination does not map *a priori* to a particular visual resolution or corresponding retinal eccentricity, we systematically blurred target images to determine the level at which haptic discrimination matched that of Experiment 1. We chose 62.5% as our performance benchmark, as the minimum (30/48 correct) corresponding to significantly above-chance performance under the binomial test we applied. In other words, that threshold served as a conservative individual-level benchmark for crossmodal echo-haptic transfer.

### Experiment 2 methods

#### Participants

Twenty-two typically sighted participants (nine female) ranging in age from 19 to 50 years (mean age 25.10 years, SD 7.74 years) completed this task. Participants provided informed consent according to protocols approved by the Smith-Kettlewell Eye Research Institute and University of Central Arkansas Institutional Review Boards, and were compensated for their participation.

#### Stimuli

**Figure 5:**
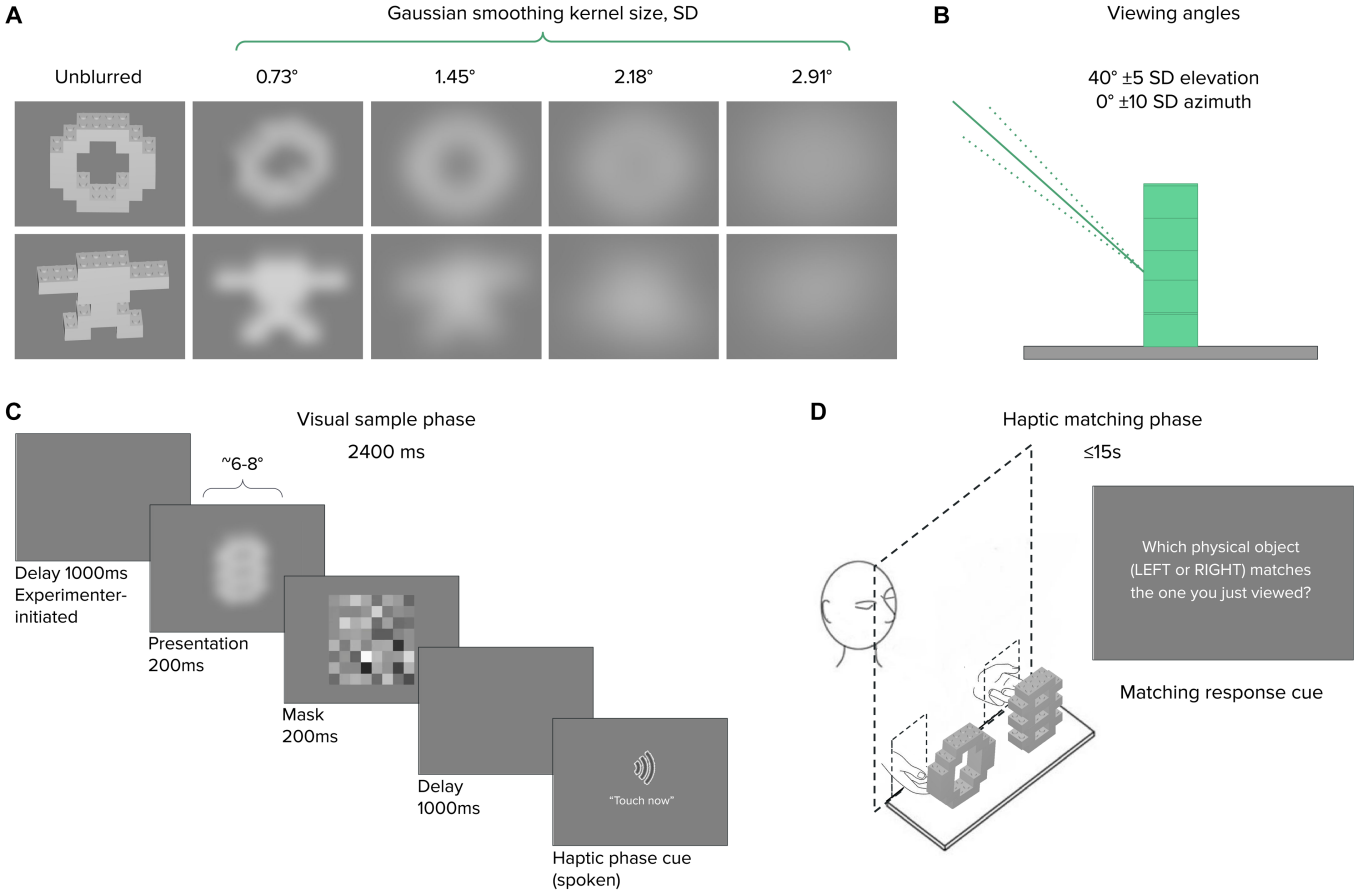
Experiment 2 stimuli and procedure. **(A)** Representative novel object image renderings shown unblurred, and convolved with 2-D blurring kernels as shown; viewing angle jitter illustrated in **(B)**. The visual sample phase **(C)** lasted 2,400 ms, followed immediately by a haptic matching phase **(D)** with an opaque occluding screen. After 15 s, a response cue prompted a LEFT/RIGHT keyboard response.

Visual stimuli comprised object images generated as follows: 3D models of each of the 16 Novel LEGO^®^ objects used in Experiment 1B were generated using the LEGO^®^ Digital Designer virtual building environment. The resulting object files were exported to Matlab (The Mathworks, Natick, MA) to generate grayscale visual renderings for presentation during the experiment. For each object, the viewing angle was selected by drawing from two normal distributions centered on a straight-on azimuth (±10° SD) and 40° elevated perspective (±5° SD), respectively. In this way, we jittered the presentation angle to approximate the average but varying pose of the Experiment 1 participants relative to the tabletop-mounted objects. Lighting was set to originate from the viewer’s perspective, and the background was a neutral gray (128/255 pixel value). Reflective surfaces typically took on ~72% of maximum pixel intensity (median 183, IQR ~175–187, see Supplemental Data for details). Next, each object was blurred using MATLAB’s *imgaussfilt* function, which applied a 2-D Gaussian smoothing kernel to the image with one of four σ values ranging from ~0.7° to ~2.9° visual angle (Figures 5A,B). The brightness distribution changed with increasing blur, but the overall range was relatively constant per object.

#### Procedure

Participants sat 57 cm away from a Viewsonic IPS LCD monitor. Before beginning each experiment, participants received verbal and written instructions from the experimenter. Each trial consisted of a visual sample phase and a haptic match phase. In the visual sample phase of each trial, a blurred object image was presented for 200 ms against a gray background, followed by a 2 s mask (Figure 5C). We chose 200 ms as a presentation duration to be sufficient for object processing within a single fixation at multiple blur levels (see Discussion for details). Object images subtended 6–8° visual angle, each blurred at 1 of the 4 levels described above, following a method-of-constant-stimuli approach. An audio verbal cue (the spoken words “touch now”) marked the onset of the haptic match phase, in which participants reached their hands through openings in an occluding board and haptically inspected two Lego objects positioned behind the occluder, one to the left and one to the right. Participants indicated the target object via button press on a computer keyboard (Figure 5D). A second auditory cue alerted participants to respond after ~15 s if they had not already done so. A session consisted of 80 trials, with each object appearing as a target 5 times, each time paired with a different distractor. To ensure that participants were able to detect the very briefly presented images during the experimental trials, we introduced a 10-trial practice block starting with the 11th participant.

#### Analysis

For each participant, we analyzed haptic discrimination performance as a function of visual blur. We estimated individual psychometric functions with the psignifit4 Matlab toolbox (Schütt et al., 2016), fitting a cumulative normal function to the data using psignifit’s beta-binomial method. The guess rate was constrained to 50% correct, with lapse rate as a free parameter. Blurring kernel size was expressed as cycles per degree (cpd) to increase along with performance. Based on our performance benchmark from Experiment 1, the threshold was set to the 62.5% intercept, independently of psignifit’s scaling of the fitted function, i.e., irrespective of the computed lapse rate. Additionally, we estimated the group-level threshold blur by computing a pooled psychometric function for all 22 participants. The resulting threshold values were inverted to obtain a measure in degrees visual angle for the blurring kernel 𝝈, doubled for an estimate of the full kernel width, or multiplied by 2.35 for full-width half-max (FWHM). To estimate equivalent visual acuity from minimum angle of resolution (acuity = 1/MAR), we follow a rule of thumb from Hogervorst and van Damme (2008) in which blur threshold 𝝈 was found to be half the MAR, thus:


$$\mathrm{acuity}=\frac{1}{2\sigma_{\mathrm{Threshold}}}$$

### Experiment 2 results and discussion

**Figure 6:**
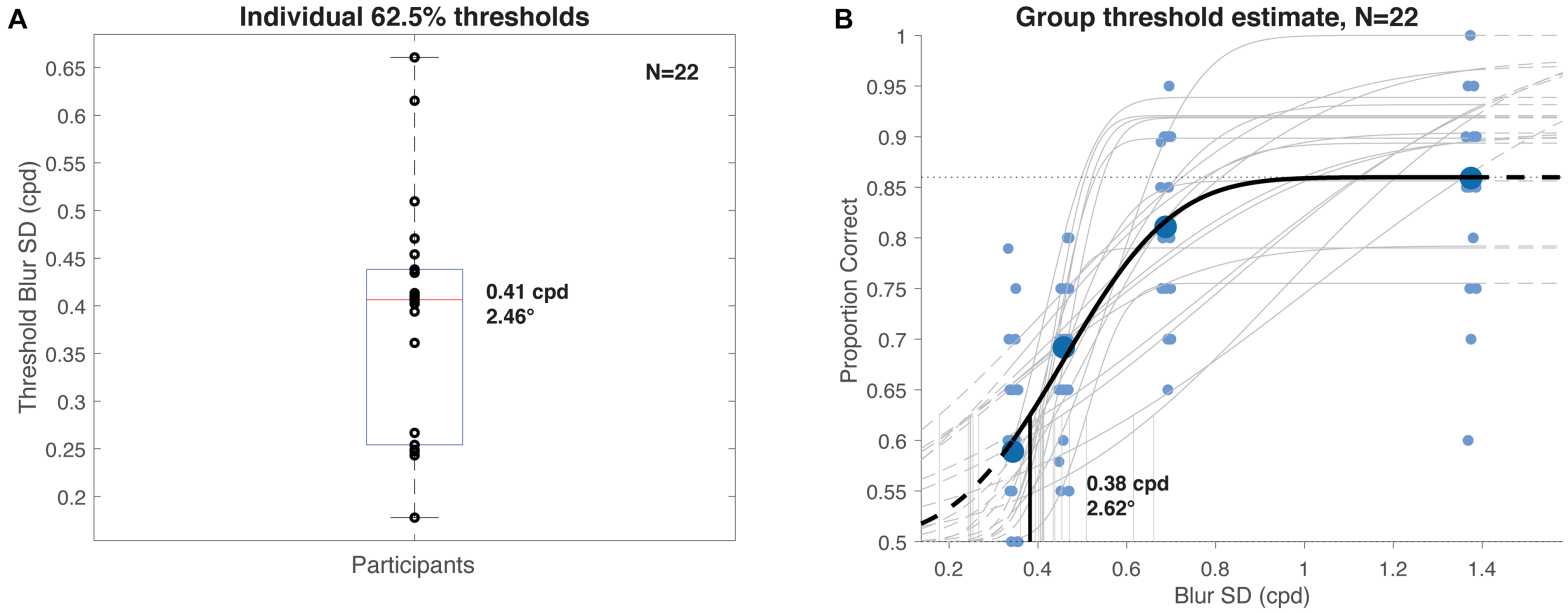
Experiment 2 visuo-haptic discrimination results. **(A)** Distribution of individual participants’ 62.5% haptic discrimination thresholds (details in main text) computed as a function of visual blurring kernel size. Median value was 0.41 cpd (~2.5°). **(B)** Group psychometric function and threshold (heavy curves) computed from pooled participant data (large data points), overlaid on individual subject estimates (gray curves, small data points, x-positions jittered for clarity). Estimated threshold value was 0.38 cpd (~2.6°).

Average performance on the visuo-haptic object discrimination task ranged from 86% in the least blurred condition, with one participant achieving 100% accuracy, to 59% for the blurriest images. The pooled psychometric function and the distribution of individual threshold estimates is shown in Figure 6. Median individual performance (Figure 6A) corresponded to a Gaussian blur with 𝝈~2.46° (±1.65° iqr), or width ~ 4.92°. The pooled threshold (Figure 6B) was 0.38 cpd, corresponding to a Gaussian blur with 𝝈~2.62° (width ~ 5.23°). These filter sizes represent estimates of the information resolution available for comparison during haptic discrimination, expressed as equivalent visual blur.

That several participants achieved 90% or greater accuracy in the two least blurred conditions suggests that object information could be extracted, retained, and crossmodally transferred to the haptic matching phase of the trial despite the brief presentation and masking of the images. The monotonic decrease in haptic performance with increasing blur level suggests that visual resolution of the sample directly affected the fidelity of the representation used for discriminating objects by touch.

## General discussion

In Experiment 1, we tested the ability of blind experts and sighted novice echolocators to identify a common or novel object haptically after sampling it echoically. Participants in both groups demonstrated at least some ability to echolocate target objects and then haptically identify them, supporting the notion that echoic object shape information can be encoded and transferred across sensory modalities. The blind group performed most consistently, with blind observers echo-haptically discriminating both object classes above chance as a group, and no participant scoring below 50%. In contrast, after correcting for multiple comparisons, sighted observers performed significantly above chance for Common but not Novel objects, although a mixed ANOVA based on the participant subset who completed both conditions did not reveal significant effects or interactions related to group or object class. Taken together, the results suggest that echolocation experience, visual status, or object familiarity may influence echo-haptic performance, even if they do not strictly gate it. The age of blindness onset did not appear to strongly predict performance in the echolocators, unlike previous work suggesting such a pattern (Teng et al., 2012), although the sample size here was too small to support a formal comparison. Notably, blind participants reported that they did not often echolocate household-sized objects in everyday life as in the study. Thus, object familiarity may itself reduce to object variability: aside from shape, the Common objects also differed along more dimensions (material, mass, height and width, density, surface angles, etc.) compared to the Novel set.

In Experiment 2, we tested the ability of sighted observers to haptically discriminate objects after briefly sampling them visually at varying levels of imposed blur. By examining individual as well as pooled thresholds, we found visuo-haptic performance to approximate that of echo-haptic performance in Experiment 1 when images were blurred with a 𝝈~2.5° kernel, or subtending about 5° (5.9° FWHM). We interpreted this result as a visually-based estimate of the equivalent spatial resolution available to the participants in Experiment 1. The strong and systematic influence of image blur on performance in Experiment 2 suggests that resolution of the sensory input during sampling, rather than the haptic matching or the crossmodal transfer process itself, is the bottleneck on haptic discrimination. This is consistent with prior evidence that sampling factors rather than the resulting internal representations underlie the crossmodal costs of object recognition (Newell et al., 2001; Ernst et al., 2007).

The principal goal of Experiment 2 was to compare echolocation and vision indirectly, by equating crossmodal haptic matching performance after sampling using each respective modality. The reasonable question arises: Can an echoacoustic task truly be characterized in “equivalent” visual terms? I.e., is it meaningful to equate haptic discrimination performance following (1) several dozen echo clicks over unconstrained time vs. (2) a blurred grayscale image flashed once for 200 ms? In principle, a practically infinite combination of manipulations and parameters could produce 62.5% haptic object discrimination performance; a different set of objects with different features would elicit a different threshold blur value. In this sense, the current study represents just one possible way echolocation could be evaluated and compared to other modalities people use to interact with the world. Other research studying crossmodal interactions also chooses certain ways and parameters to equate across modalities. For example, blurring a visual target past 60° width reverses the classic auditory–visual ventriloquist effect (Alais and Burr, 2004), which is a meaningful threshold result even if different stimulus parameters would have changed the specific value.

Though representing merely a subset of the reasonable possibilities, the stimuli, task constraints, and visual presentation conditions used here are consistent with the way object perception (in the respective modalities) has been studied in the laboratory and observed in everyday life. Furthermore, a 200ms visual presentation duration, falling within typical first-saccade latencies (Rayner, 2009; Võ and Henderson, 2010; Cronin et al., 2019), permits object recognition even at significant blur levels (Kwon et al., 2016). Additionally, ~20–60 clicks per trial, at ~5 ms per click-echo pair, yields ~100–300 ms of total echoacoustic stimulation. Though these coarse estimates do not directly match echo vs. visual conditions, they suggest that information obtained from the objects sampled in Experiments 1 and 2 was comparable enough to match meaningfully via a third shared modality.

### The challenges of echolocating objects vs. space

Despite the uncertainties inherent in crossmodal estimates of equivalent visual resolution, our results provide a starting point for estimating the spatial grain of object echolocation. A blur threshold of 2.5°, converted to minimum angle of resolution, corresponds to a visual acuity of about 0.2, falling within low-vision acuity regimes measured in older observers with, e.g., diabetic retinopathy and macular degeneration (Hogervorst and van Damme, 2008). By contrast, we previously measured echolocalization thresholds as fine as 1.5° (Teng et al., 2012), corresponding roughly to visual letter recognition thresholds at 35° retinal eccentricity (Anstis, 1974) or a converted visual acuity of ~0.33 (Hogervorst and van Damme, 2008). For comparison, blur thresholds for purely visual form recognition are less than 10 arcmin for untimed displays (Westheimer, 2013). For presentation times matching those in our study, estimated thresholds fall within a factor of 2 to 3 of our reported values (Kwon et al., 2016).

For both Common and Novel object sets, overall echo magnitude may have varied between object exemplars, but not in a reliably systematic way, unlike previous studies where object size (Teng and Whitney, 2011) or shape (Thaler et al., 2011; Arnott et al., 2013) likely correlated with overall echo magnitude. Not necessarily as described above, many factors make any two given scenarios difficult to compare directly. However, if objects are similar in overall size, shape, and location (Rosenblum et al., 2000; Milne et al., 2014), shape differences would be indexed by complex echoacoustic features defining 2- and 3-dimensional shape, i.e., the spatiotemporal “acoustic image” of object structure which would be perceived by bats and dolphins (Simmons, 1989; Herman et al., 1998) but less accessible to the comparatively lower-frequency echoes and longer integration times (Au et al., 1988; Muchnik et al., 1991; Vanderelst et al., 2016) of human echolocators’ auditory systems. To overcome this difficulty, observers in the present study may have indexed object differences by integrating echo returns across head/body positions, a “proprioceptive analogue” to anorthoscopic perception (Rock, 1981; Orlov et al., 2021) consistent with prior evidence of perceptual benefits to self-motion and substantial “penalties” for fixed positions (Rosenblum et al., 2000; Milne et al., 2014).

In a recent computational study (Christensen et al., 2020), a generative neural network was trained to associate binaural echoacoustic recordings with concurrently recorded stereo grayscale images, then to predict the grayscale and depth maps from the echoes alone. The model most successfully reconstructed scenes in which spatial information varied smoothly and systematically, e.g., a view directly along an empty hallway, suggesting that the spatial layout of a scene transfers readily between visual and echoacoustic modalities. By contrast, the model largely failed to reconstruct visually cluttered scenes with complex objects (e.g., office chairs) from binaural scene echoes. In similar fashion, an equivalent blur estimate, while not directly analogous to the sophisticated acoustic image of non-human echolocators, could predict echolocation performance in visual form in novel situations, e.g., in individualized scenarios essential to orientation and mobility training (Wiener et al., 2010).

### Should visual metrics be used to benchmark echolocation?

Importantly, we do not purport to hold equivalent visual acuity/blur up as an immutable metric to evaluate all nonvisual perception. (It cannot even do that for vision, as it misses deficits from, e.g., various ocular (Xiong et al., 2020) and non-ocular (Good et al., 2001) vision disorders.) The final arbiter of any perceptual mode—whether vision, audition, haptics, echolocation, or any technically mediated versions thereof—should be its functional utility for the observer rather than an arbitrary acuity metric (Colenbrander, 2010; Striem-Amit et al., 2012). Nonetheless, standardized measures are useful to establish a common frame of reference, provide a sense of the spatial bandwidth available to an observer, bound the expectations for training interventions, and inform benchmarks for the design of assistive technologies. For example, to illustrate the difficulties faced by sensory-substitution devices (SSDs) for blind users, a previous study (Loomis et al., 2013) estimated a 500-fold bandwidth advantage for vision vs. touch, based on relative resolutions and fields of view between the retina and the fingerpads of one hand. Another (Striem-Amit et al., 2012) estimated equivalent visual pixels perceived by congenitally blind visual-to-auditory SSD users in a Snellen acuity task, in some cases exceeding the World Health Organization acuity threshold for legal blindness. In our case, we were motivated in this and previous (Teng et al., 2012) work to concretely address questions like “How well can you echolocate?”—a deceptively simple query that, until recently, has lacked a well-developed framework for answers.

### Practical implications

Our results and approach suggest some promising avenues for researchers and practicing echolocators to understand and optimize echoacoustic object perception. First may simply be for a practitioner to focus their efforts on ensonifying larger objects than our tabletop-sized stimuli. We chose the stimulus sets in the present study because they spanned the size and shape of commonly manipulated everyday objects, as well as objects previously discriminated crossmodally by dolphins (Harley et al., 2003) or bats (Danilovich and Yovel, 2019). Obstacle-level objects, such as furniture or larger appliances, are likely to have more spatially extended features accessible not only to experts, but even to sighted echolocators with modest amounts of training (Teng and Whitney, 2011; Norman et al., 2021). At these scales, more robust acoustic object cues as well as group behavioral differences may emerge. Second, one can leverage the greater acuity of azimuthal echolocalization (or, conversely, circumvent its absence as a cue) to assist object form perception via self-motion to incorporate proprioceptive and positional cues (Milne et al., 2014). Third, increasing the effective resolution of the emitted signal should not only aid spatial/navigational echo perception but make objects more distinctly perceptible. For example, assistive devices that record and slow down ultrasonic echoes (Ifukube et al., 1991; Sohl-Dickstein et al., 2015; Sumiya et al., 2019) can make the fine temporal structure of acoustic images more available to human perception, with the added benefit of greater spatial resolution afforded by ultrasound. Assessing the effectiveness of such a device could entail an equivalent acuity estimate for object discrimination with ultrasonic echoes, straightforwardly comparable to the present results.

## Data availability statement

The raw data supporting the conclusions of this article will be made available by the authors, without undue reservation.

## Ethics statement

The studies involving humans were approved by the UC Berkeley Committee for Protection of Human Subjects, the Smith-Kettlewell Institutional Review Board, and the University of Central Arkansas Institutional Review Board. The studies were conducted in accordance with the local legislation and institutional requirements. The participants provided their written informed consent to participate in this study.

## Author contributions

ST: Conceptualization, Data curation, Formal analysis, Funding acquisition, Investigation, Methodology, Project administration, Resources, Software, Supervision, Validation, Visualization, Writing – original draft, Writing – review & editing. CD: Funding acquisition, Investigation, Project administration, Resources, Writing – original draft. NP: Investigation, Methodology, Project administration, Resources, Software, Writing – original draft. ME: Funding acquisition, Methodology, Software, Visualization, Writing – original draft. AP: Conceptualization, Funding acquisition, Investigation, Methodology, Project administration, Resources, Software, Supervision, Validation, Visualization, Writing – original draft, Writing – review & editing.
