# Supplementary material for: Object recognition via echoes: quantifying the crossmodal transfer of three-dimensional shape information between echolocation, vision, and haptics
Source: Front Neurosci. 2024 Feb 19;18:1288635. doi: 10.3389/fnins.2024.1288635 (PMC10909950; doi:10.3389/fnins.2024.1288635)
Supplement: Supplementary file 1 [file Table_1.DOCX]

Teng et al. — Appendix: Stimulus objects


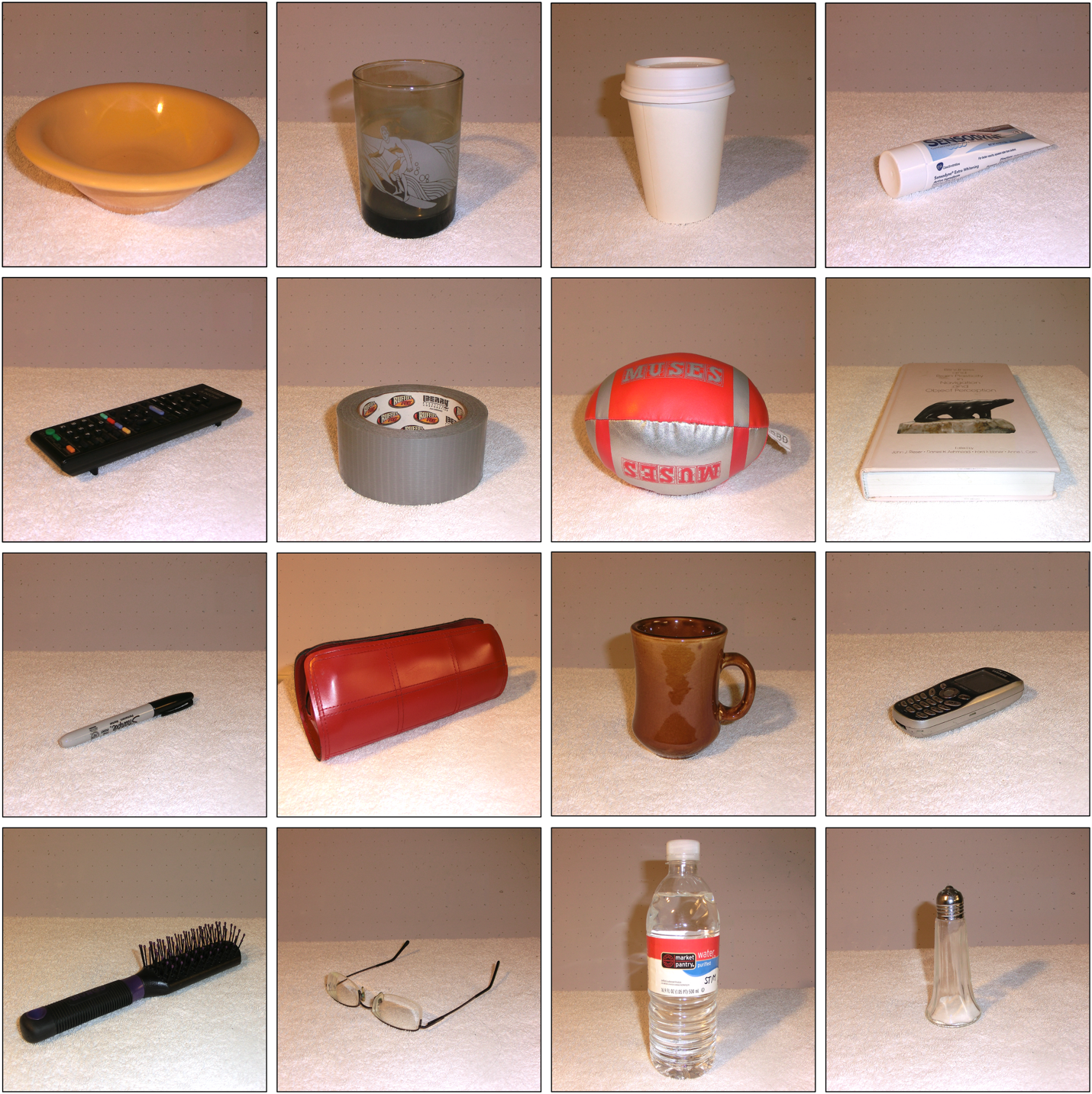


Figure S1. Common household objects. Width of each image frame spans approx. 20cm.


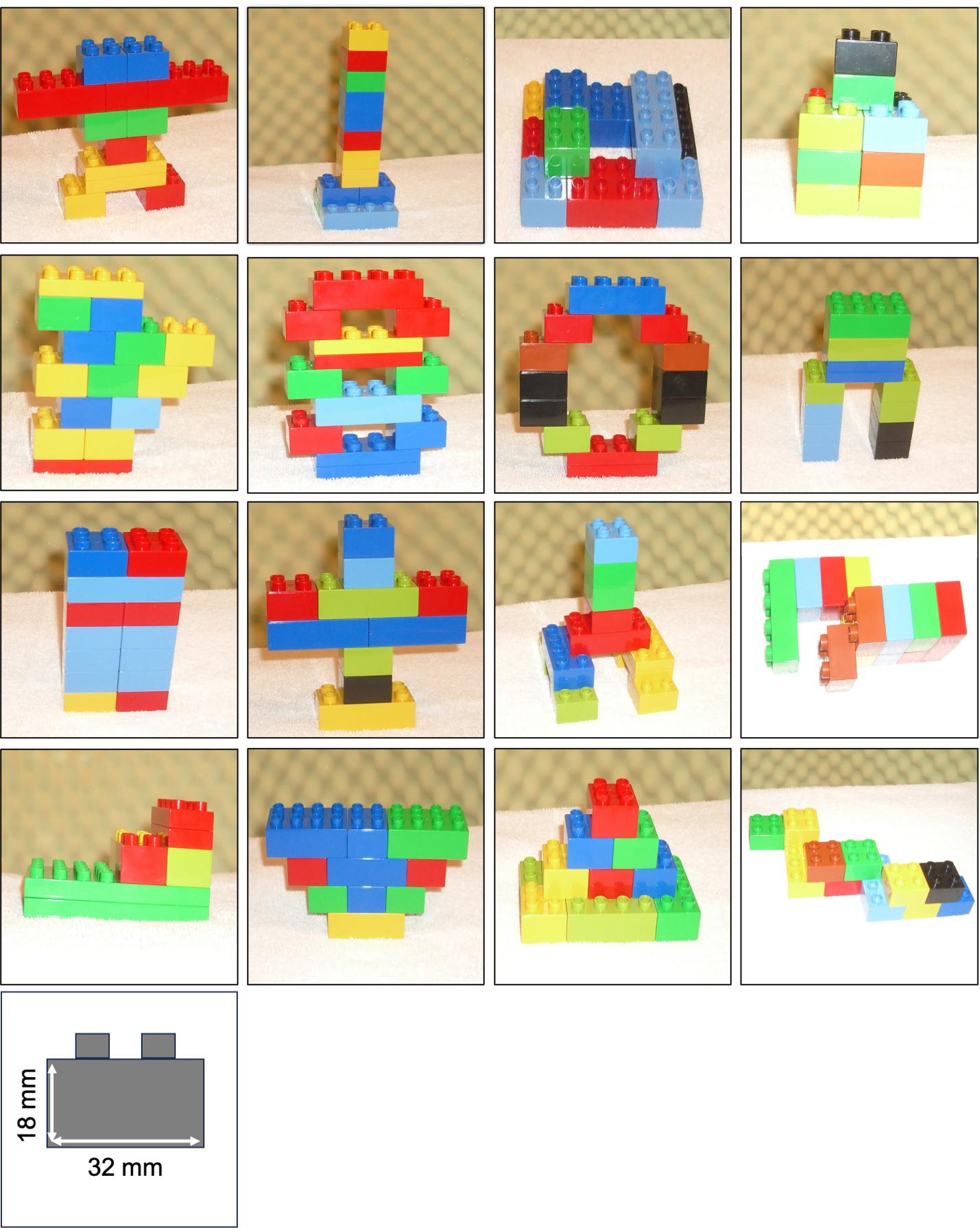


Figure S1. Novel LEGO^TM^ objects. Dimensions for 2-stud standard block shown in bottom panel.
