# Supplementary material for: Object recognition via echoes: quantifying the crossmodal transfer of three-dimensional shape information between echolocation, vision, and haptics
Source: Front Neurosci. 2024 Feb 19;18:1288635. doi: 10.3389/fnins.2024.1288635 (PMC10909950; doi:10.3389/fnins.2024.1288635)
Supplement: Supplementary file 2 [file Data_Sheet_1.PDF]

Supplemental data: Computing visual pixel intensities for Experiment 2 stimuli

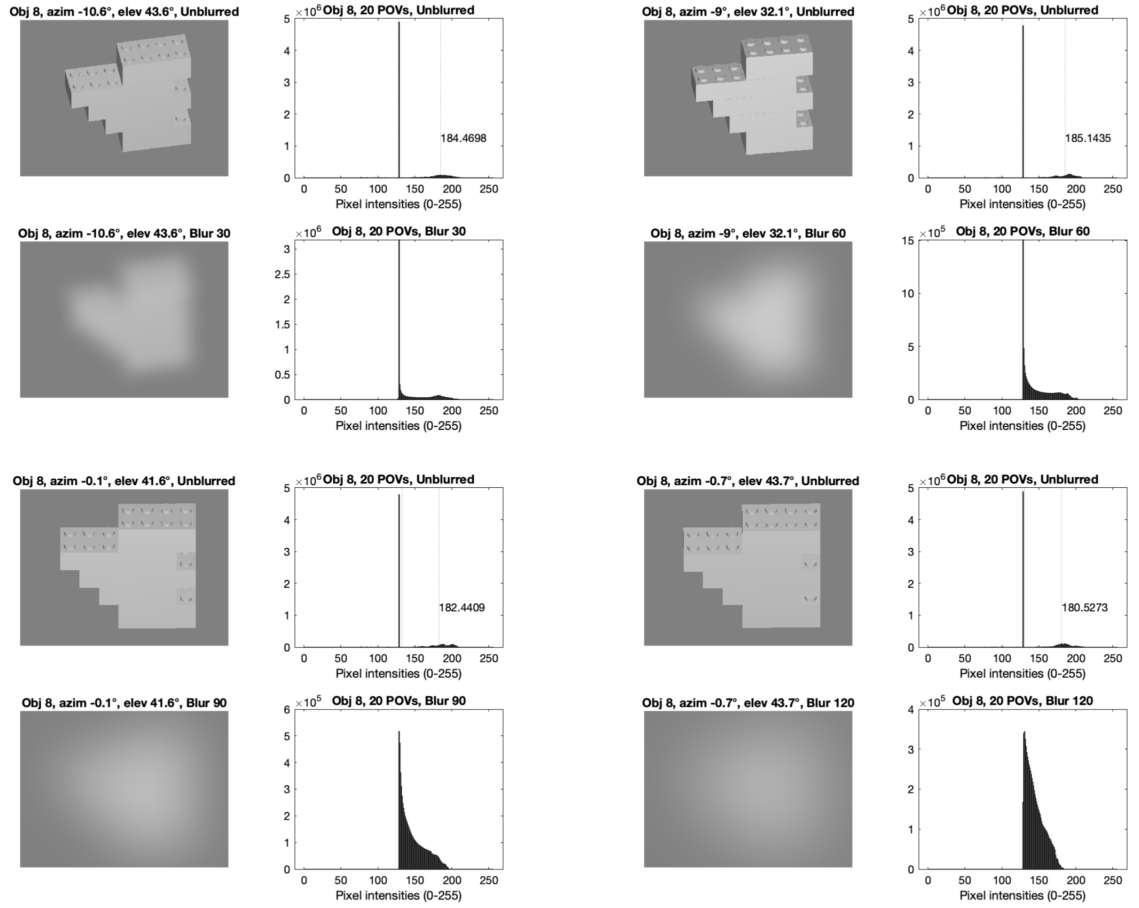

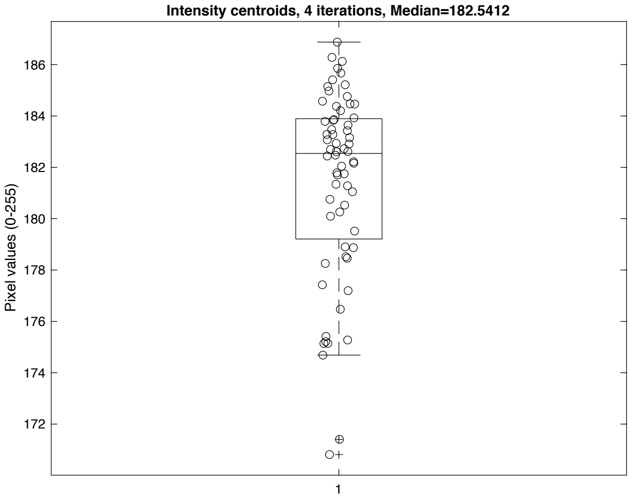

Top figure: For every object, we computed 20 jittered viewing angles from the distribution described in the text. We then pooled the pixel intensity histogram across all 20 viewing angles to "smooth out" incidental visual features specific to any particular angle. The result was a bimodal intensity distribution for each object with a spike at 128 (the background gray) and a cluster of brighter values indicating surface reflections. Next, we performed a k-means cluster analysis of the pooled distribution with k=2. One centroid was always the background gray, and the other was always the cluster of brighter values. For illustration, each pooled histogram is paired with the object image, oriented at one randomly selected viewing angle. Vertical dotted line and annotated value denote centroids in unblurred histograms. Multiple blur levels shown; note that the distribution changes with blur, but the range of values remains constant. **Box/scatterplot figure**: Distribution of centroid values across 16 objects, iterated 4 times each (only unblurred images were analyzed in this fashion).
